# Complexity as a factor for task allocation among general practitioners and nurse practitioners: a narrative review

**DOI:** 10.1186/s12875-020-1089-2

**Published:** 2020-02-17

**Authors:** Robyn Cody, Stefan Gysin, Christoph Merlo, Armin Gemperli, Stefan Essig

**Affiliations:** 1 Institute of Primary and Community Care, Schwanenplatz 7, 6004 Lucerne, Switzerland; 2 Department of Health Sciences and Health Policy, University of Lucerne, Lucerne, Switzerland; 3 Swiss Paraplegic Research, Nottwil, Switzerland

**Keywords:** Narrative review, Collaborative practice, Interprofessional collaboration, Task sharing, Primary care, Nurse practitioner, General practitioner, Family medicine, Complexity, Task allocation

## Abstract

**Background:**

General practitioner (GP) shortages and increasing demand for care led to the introduction of nurse practitioners (NPs) to primary care. Many concepts for task sharing among health professionals feature complexity. The aim of this narrative review was to examine how complexity is used as a factor for task allocation between GPs and NPs.

**Methods:**

According to the PRISMA statement, PubMed and CINAHL were searched systematically, and eligibility criteria were applied to detect literature concerning GPs and NPs in primary care and complexity in the context of task allocation. Relevant information was extracted, and a narrative analysis was performed.

**Results:**

Thirty-seven studies from seven countries were included, comprising quantitative, qualitative, and mixed methods. Complexity was used to describe patients, their needs, and health professionals’ tasks. The understanding of the use of complexity as a factor for task allocation between NPs and GPs was based on the patient population (specific vs. unspecific), the setting (specific vs. unspecific), the numbers of health professionals involved (two vs. more than two), and the NP role (distinct model of care vs. no model). Despite similarities in these areas, the tasks which NPs perform range from providing minor to complex care. However, there is a slight trend towards NPs treating socially complex patients and GPs focusing on medically complex cases.

**Conclusion:**

Complexity as a concept is prominent in primary care but remains broad and inconsistent as a factor for task allocation between NPs and GPs. This review can be used as a point of reference when practitioners are seeking methods for task allocation in a collaborative primary care setting.

## Background

In an era of a world-wide general practitioner (GP) shortage and increased demand for health care services because of chronic illness and ageing, evidence shows that 25–70% of physician tasks could be delegated to non-medical health professionals in advanced roles, especially in primary care [1]. Introducing additional and varied professions into primary care has been deemed an appropriate solution to counteract this shortage while addressing the increased need for primary care services [2–4].

Evidence shows that nurses are capable of independently conducting 85% of GP same day appointments [5], providing as high a quality of care and achieving equivalent health outcomes as GPs [6], and contribute to reducing hospitalisations and mortality rates [7]. Particularly nurse practitioners (NP) invoke high levels of patient satisfaction [8, 9]. The titles, training, and experience of NPs vary greatly internationally, leading to them working in various fields and with varying scopes of practice [10]. According to the International Council of Nurses (ICN) “a Nurse Practitioner/Advanced Practice Nurse is a registered nurse who has acquired the expert knowledge base, complex decision-making skills and clinical competencies for expanded practice, the characteristics of which are shaped by the context and/or country in which s/he is credentialed to practice. A master’s degree is recommended for entry level.” [11]

Introducing interprofessional teams results in the need for task re-allocation. This can be done using the concept of skill mix in which professionals with different qualifications collaborate, emphasizing the utilization of professional’s knowledge, experience, and skills to their fullest potential [1]. Alternatively, allocating tasks according to the principle of subsidiarity can lead to an imbalance in workload and dissatisfaction among health professionals, thus perpetuating staffing issues [12]. Evidence to date suggests that the concept of complexity features when allocating tasks in primary healthcare teams and bears a noteworthy impact on interprofessional collaboration [13, 14]. When looking for definitions of complexity within medicine, an evolution of the term has been discovered. Surrogate terms such as comorbidity, multimorbidity or polypathology were often used to describe what today, may be referred to as complex. These terms all refer to a multitude of conditions and or diseases [15]. According to a concept clarification “complexity, as opposing to the previous surrogate terms [sic!], promotes a wider perspective of health by expanding the focus on biology to include the environment and social relations.” [15](p.18) Complexity can also be regarded as a system within which people act. As shown in the Cyenfin Framwork [16] which is based on people operating in one of four systems: simple, complicated, complex or chaotic. In an adaptation which divides various medical fields in to the four systems it is said that holistic medicine represents a complex system. In this model holistic medicine is characterized by informal and interdependent care in which experienced practitioners rely on narratives and metaphors to recognize patterns and make sense of complexity in order to act [17]. Furthermore, complexity can be regarded as a concept according to which professional tasks are allocated, as shown in Kernick’s continuum [18]: the higher the complexity the more educated the health professional. Health professionals range from A to E. A being a GP managing and planning the treatment of patients based on the interpretation and integration of complex clinical, psychological, social, cultural and cost factors in combination with experience and knowledge. Addtionally organizing and coordinating multidisciplinary teams. B being a NP clinically diagnosing and treating less complex cases, active in some areas of chronic care while interacting with other members of the team. C being an Extended Role Practice Nurse providing specific, well-defined, protocol-directed clinical care, for example asthma or contraception management. D being a Practice Nurse providing traditional nurse care, for example the management of minor injuries or immunization. Finally, E being an auxiliary Practice Nurse with limited training performing simple, well-defined tasks such as urine analysis or simple wound dressings [18]. Allocating tasks according to this continuum based on complexity suggests a shift from very separate, different nurse and doctor roles towards a partnership which is inherently flexible. Additionally, when looking at task distribution on a continuum, it is possible to make the most of each professionals’ skills and time thus ensuring health gain in an effective and economic way according to Kernick. The premise of this continuum is, that the less training, the less responsibility and complexity and also the less remuneration.

Despite the level of importance assumed by the concept of complexity in existing literature and the theoretical constructs, to date there is no overview, which provides practical guidance for practitioners on the precise use of complexity as a factor for task allocation. By looking at recently published studies in which NPs have been introduced into primary care and are collaborating with GPs the use of complexity can be examined and insights into possible methods for task allocation gained. Additionally, information regarding tasks performed by NPs working collaboratively in different primary care settings and countries may contribute to understanding the role of NPs further. This may be particularly helpful for practices seeking to implement or enhance skill mix. Therefore, the objective of this narrative review was to investigate the reported use of complexity as a factor for task allocation among GPs and NPs working collaboratively in primary care by collecting and analysing existing evidence based on quotes referring to complexity.

## Methods

A protocol was written to guide the methodological process following the PRISMA statement [19]. Evidence pertaining studies set in primary care and describing the collaboration between NPs and GPs were searched for in scientific databases. This was deemed an appropriate method of reaching the goal of creating an overview of how complexity is used in task allocation in models of primary care which offer some model of shared care. Literature was examined in a broad manner with the goal of linguistically detecting the term complexity or related terms and analysing the context.

### Information source and search strategy

Database searches were carried out in PubMed and CINAHL in November 2019 using search terms built upon three concepts: nurse, role, and GP. For the search in PubMed the terms comprised Medical Subject Headings (MeSH) and free text words combined using Boolean operators and truncations as seen in Table 1. Here, the time and language restrictions were included in the search terms. For the search in CINAHL, MeSH terms were replaced with Exact Subject Headings (MH). Additional filters were put in place as follows: scholarly journals, published dates: July 2006 – November 2019, languages: English and German. Forward cited literature and bibliographies of the resulting literature were searched manually to complete the selection.

**Table 1:** Search strategy

Concept 1: Nurse	
1 Nurse [All Fields]	
2 “registered nurse*”[All Fields]	
3 “clinical nurse*”[All Fields]	
4 “Nurse Practitioner*”[All Fields]	
5 “Nurse Practitioners”[Mesh]	
6 “Advanced Practice Nurse*”[All Fields]	
7 “Advanced Practice Nursing”[Mesh]	
8 “Advanced nursing practice”[All Fields]	
9 “Public health nurse*”[All Fields]	
10 “Nurses, Public Health”[Mesh]	
11 “Community nurse*”[All Fields]	
12 “Nurses, Community Health”[Mesh]	
13 “Nurse Clinicians”[Mesh]	
14 “Family Nurse Practitioners”[Mesh]	
15 “Nurses, International”[Mesh]	
OR 1–15	
Concept 2: Role	
16 Task*[All Fields]	
17 “Task Performance and Analysis”[Mesh]	
18 “Scope of Practice”[All Fields]	
19 Role*[All Fields]	
20 “Nurse’s Role”[Mesh]	
21 “Physician’s Role”[Mesh]	
22 interprofessional [All Fields]	
23 “Interprofessional Relations”[Mesh]	
24 Cooperation [All Fields]	
25 “Cooperative Behavior”[Mesh]	
26 Collaboration [All Fields]	
27 Team*[All Fields]	
28 “Patient Care Team”[Mesh]	
29 Teamwork [All Fields]	
30 “Skill mix”[All Fields]	
31 “Staff mix”[All Fields]	
32 “Integrated care”[All Fields]	
33 “Delivery of Health Care, Integrated”[Mesh]	
34 “Practice Patterns, Nurses’”[Mesh]	
35 “Practice Patterns, Physicians’“[Mesh]	
36 “Physician-Nurse Relations”[Mesh]	
OR 16–36	
Concept 3: GP	
37 “General Practitioner*”[All Fields]	
38 “General Practitioners”[Mesh]	
39 “Primary Care Physician*”[All Fields]	
40 “Physicians, Primary Care”[Mesh]	
41 Doctor [All Fields]	
OR 37–41	
Exclusion	
42 “Hospitals”[Mesh]	
43 hospital [All Fields]	
OR 42–43	
Formality: Time	
44 “2006/07/01”[PDAT]: “2019/11/30”[PDAT]	
Formality: Language	
45 English [lang]	
46 German [lang]	
OR 45–46	
Combining	
(1-15OR) AND (16-36OR) AND (37-41OR) NOT (42-43OR) AND 44 AND (45-46OR)	

OR, AND, NOT = Boolean operators,

*MeSH* Medical subject heading

### Eligibility criteria

There are two sets of eligibility criteria, which can be seen in Table 2. Stage 1 criteria were applied to assess titles and abstracts and stage 2 criteria to assess full texts. There were no restrictions regarding the study type because the concept of complexity is not bound to a specific study design. Furthermore, it was unclear how much literature would suit the inclusion criteria and therefore, imposing minimal restriction led to a comprehensive search of all up-to-date literature.

**Table 2:** Eligibility Criteria

Stage 1 Titles and Abstracts - Exclusion		
Formal	Professionals	Setting
Language other than English or German	No nurse	Unspecified / Multiple
Outside time range July 1st 2006 - November 30th 2019	In professional training	Hospital / Rehabilitation centre or clinic
Lay journals, unobtainable full texts	Specialised multidisciplinary physicians	Nursing homes / Community dwellings
		Specialised outpatient clinics
		Specialised services
Stage 2 Full Texts - Inclusion		
Terms	Context	Professional
Complex, difficult, minor, easy	Terms in the context of task allocation	NP

*NP* Nurse practitioner

Stage 1 criteria stated that abstracts in a language other than English or German, published outside the range of July 1st 2006 to November 30th 2019, of non-scientific articles and opinion papers, featuring no nurse, a professional in training or specialised multidisciplinary physician, in a setting other than primary care must be excluded. Languages had to be restricted to those that the authors could understand without the need of a translator because funding was limited. Furthermore, NPs are predominantly established in English-speaking countries and in countries that mainly publish in English, e.g., the Netherlands. The time frame was chosen to include up-to-date concepts applied in current health care systems.

Stage 2 criteria stated that the full text must explicitly mention NPs. The rationale for focusing on NPs was because it is a term widely used to describe advanced nursing roles who may have the potential not only to practice collaboratively but also independently within a team. Hence, the possibility for NPs tasks to differ from GPs in complexity warrants further investigation. Additionally, at least one of the terms: complex, difficult, minor or easy must explicitly be mentioned in the context of task allocation. These terms were validated by conducting a search with multiple synonyms and antonyms for complexity (complex, complicated, intricate, difficult, simple, easy, uniform, and minor). Two random samples of 50 studies each were searched for all synonyms to evaluate which ones would yield the studies relevant to the research question. Studies containing the terms complicated, intricate, simple and uniform were excluded, as they did not provide relevant results. Lastly, the relevant text passages had to be part of the studies’ own findings and not part of a reference to another study. Only if the defined terms were present in the correct context was the full text read and considered for inclusion.

### Study selection

The study selection was carried out by two independent reviewers as follows: Upon completing the database searches the resultant studies were transferred into the reference manager EndNote© and de-duplicated according to the guidelines by Bramer et al. [20]. Then the application of the eligibility criteria took place. The search tool in Adobe Acrobat Reader DC© was used when applying Stage 2 eligibility criteria before the eligible full texts were read. The same process was applied to the resultant forward cited literature. Once the process was completed the reviewers compared their results. If a study was excluded or included differently, the study was discussed with a third reviewer until a consensus regarding its allocation was reached.

### Data collection process and narrative analysis

An initial, random sample of five included studies was selected for the development of an extraction sheet. Once the data extraction sheet was adapted sufficiently the included studies were reviewed systematically. Firstly, familiarization with the included studies took place and quotes in which complexity featured were located and extracted. Secondly, information to summarise the use and narrow context of complexity was gathered; in the narrow context only information directly from the paragraph in which complexity was used was taken into account. Thirdly, similarities and differences across studies were recognized in the broad context; in the broad context the entire publication was taken into account. The third step was an iterative process based on a narrative analysis following the Cochrane Consumers and Communication Review Group Guidelines [21]. The narrative analysis was chosen because a meta-analysis was not possible as the data stem from a wide range of study designs and capture various interventions as well as non-interventions, which are not conducive to being pooled and analysed. To support the narrative analysis the “Guidance on the Conduct of Narrative Synthesis in Systematic Reviews” [22] was consulted.

## Results

### Study selection

**Fig. 1:**
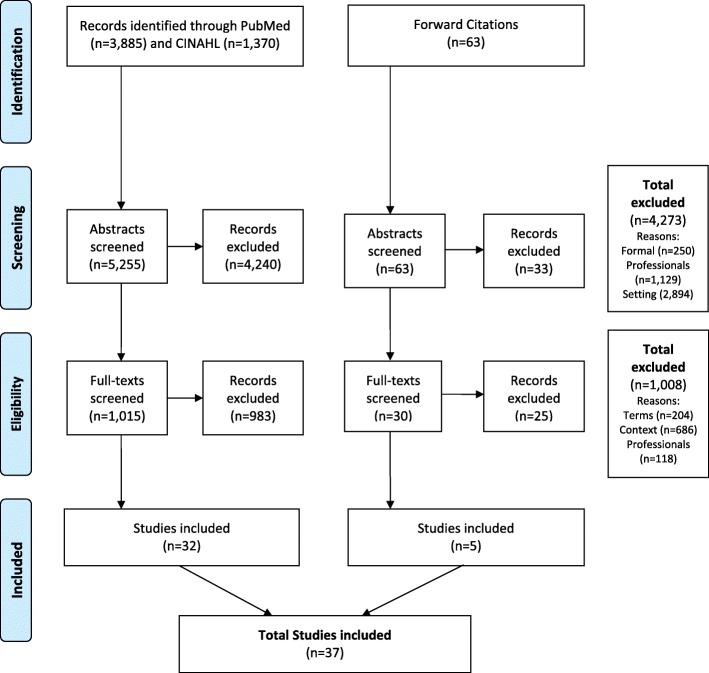
PRISMA 2009 Flow Diagram (overall)

As shown in Fig. 1 representing the PRISMA flow diagram the database searches delivered 5255 studies upon de-duplication. Titles and abstracts were screened which resulted in 4240 studies being excluded. Whereupon 1015 full texts were screened resulting in a further exclusion of 983 studies, leaving 32 studies for inclusion. During data extraction, a further 63 forward cited studies were obtained. The same process was performed, which resulted in an exclusion of 33 abstracts and 25 full texts, leading to the additional inclusion of a further five studies. Finally, 37 studies were included.

### Study characteristics

As can be seen in Table 3 the included studies date from 2007 [53] to 2019 [28–30, 52, 60]. Twenty-three are from the US [23, 25, 31–35, 37–40, 42–46, 48, 50–53, 55, 58], two from Australia [24, 57], five from the Netherlands [26, 27, 49, 54, 56], one from Norway and Finland [28], five from Canada [29, 30, 36, 41, 59] and one from the UK [60]. Eight studies are qualitative in design [23–30] and twenty-one are quantitative [31–45, 47–52]. Additionally, there are four studies in which mixed methods are applied [53, 54, 59, 60], one review [55], one country comparison [56], one case study [57], and one perspective [58]. As also shown in Table 3, complexity is used to describe patients (cases, populations, individuals, patient panels) in twenty-three studies, their needs and conditions (problems, complaints) in twenty-two studies, and health professionals’ tasks in five studies.

**Table 3:** Summary of included studies with regard to complexity

Source	Use	Context
Qualitative Study Design		
O’Brien et al. [23], US	complex medical issues	GPs take leadership with complex medical issues while NPs have a different focus.
Parker et al. [24], AU	complex medical concerns	GPs care for more complex medical conserns while NPs treat minor ailments.
O’Malley et al. [25], US	complex (care) needscomplex patients	Offloading tasks enables physicians to care for patients with complex needs. NCM also work with complex patients.
Van der Biezen et al. [26], NL	complex patientscomplex caseloadscomplex complaints	NPs enable GPs to focus on and have more time for complex patients leading to a more complex caseload while NPs treat less complex patients.
Lovink et al. [27], NL	complex care complex patients	GPs and NPs report that NPs are competent to perform geriatric assessments in older adults with complex care needs. However, the introduction of NPs in general practice means that GPs focus on more complex patients.
Boman et al. [28], NO/FI	complex care needs	NPs act as case managers especially for patients with complex care needs and comorbidity.
Côté et al. [29], CA	complex patients	GPs report their case loads including more medically complex patients upon collaborating with NPs who care for less medically vulnerable patients.
Pelletier et al. [30], CA	complex health situations complex cases	NPs enable GPs to manage more chronically ill patients while treating minor medical problems.
Quantitative Study Designs		
Ohman-Strickland et al. [31], US	complex patients	Practices with NPs could improve efficiency and individualisation of care because physicians could care for complex patients while NPs could introduce preventative approaches.
Everett et al. [32], US	complexity of populations	Populations served by NPs and doctors do not differ in complexity.
Subramanian et al. [33], US	decision-making complexity complex patients	NPs working independently with delayed physician supervision care for patients with high decision-making complexity.
Yarnall et al. [34], US	complex medical care issues	NPs can expand amount of time available for patients and free up physician’s time for complex medical care.
Chung et al. [35], US	complex conditions complex patients	NPs enabled practices to contentrate on and provide appropriate care to complex patients thus reducing the number of referrals to specialists.
Mian et al. [36], CA	complex care	FPs refer patients to NPs who serve as substitutes for less complex care.
Morgan et al. [37], US	complexity scorespatient complexity medically complex complex patients	Despite NPs and physicians having a similar complexity score, indicating NPs do treat complex patients, according to patient encounters physicians treat slightly more complex patients.
Donelan et al. [38], US	complex cases complex chronic conditions	Double the amount of physicians compared to NPs report that physicians treat more complex cases. A third of physicians report that NPs provide services for complex conditions.
Everett et al. [39], US	complex patients	NPs in a supplementary role who do not treat complex patients have similar or better outcomes compared to physician-only care. Whereas NPs in a supplementary role who do treat complex patients have worse outcomes than physician-only care.
Everett et al. [40], US	socially complex patients clinical complexity complex patients	Compared to physicians, NPs as usual providers treat more socially complex patients and similar clinical complexity. However, NPs as usual providers refer patients to physicians significantly more often than the reverse.
Dahrouge et al. [41], CA	complex medical conditions medical complexity medically complex patients socially complex patients	FPs care for patients with more complex medical conditions. NPs care for patients with less medical complexity to minimise consultations with family physicians. Compared to FPs, NPs treat more socially complex patients.
Ku et al. [42], US	complexity of care complex visits complex conditions	Pysicians are more involved in complex visits and overall involved in the care of patients with complex conditions compared to NPs.
Kuo et al. [43], US	medically complex individuals	NPs recognise limitations when treating medically complex individuals. Overall NPs may treat less medically complex patients than PCPs.
Park [44], US	complex cases	NPs in practices can increase accommodation and care coordination of patients because the physician’s time is freed up for complex cases.
Reckrey et al. [45], US	complex medical and psychosocial needs complex care situations complex patients	Team-based models of care are needed to treat complex medical and psychosocial needs. NPs take an enhanced role in the management of the most complex patients in team approach physician panels.
Marcum et al. [46, 47], US	complex patient panels	PCPs prescribe more medication on account of their patient panel being more compelx than that of NPs.
Raji et al. [48], US	complex health conditions	A team approach including NPs and MDs may be best for patients with complex health conditions.
Van der Biezen et al. [49], NL	minor ailments task complexity	NPs treat a patient panel with minor ailments.
D’Afflitti et al. [50], US	complex patients	NPs cared reach out to medically and socially complex patients to engage them in care.
Yang et al. [51], US	medical complexity	NPs caring for diabetic patients in the VHA treat similar medical complexity as GPs.
Morgan et al. [52], US	complex patients	NPs provide care for medically complex patients without increasing costs.
Other Study Designs		
Fletcher et al. (mixed methods) [53], US	complex patients complex cases complexity of patients	NPs can increase access for more patients and free up physician’s time for complex patients. In physician’s opinion NPs can only treat complex cases under constant, direct physician supervision. In physician’s opinion NPs should treat low complexity patients.
Dierick-van Daele et al. (mixed methods) [54], NL	minor health problems complex care	NPs should care for common complaints and minor health problems freeing up GP’s time for patients with chronic diseases and multimorbidity. According to a direct quote from one GP complex care is best shared by a GP and a NP.
Sustaita et al. (review) [55], US	complex patients	NPs can free up physicians’ time by taking over routine tasks and allowing physicians to treat complex patients.
Freund et al. (comparison) [56], NL	complex presentations (minor illnesses)	NPs in the Netherlands are responsible for clinical diagnosis and treatment of less complex presentations and chronic care management.
Helms et al. (case study) [57], AU	complex conditionscomplex chronic diseases	The NP has his own caseload and receives referrals from other team members according to expertise and interest. The NP increased GP productivity by caring for patients with complex diseases.
Bodenheimer & Bauer (perspective) [2, 58], US	complex health care needs	When GPs and NPs collaborate, GPs will lead the team caring for people with complex health care needs.
Hunter et al. (mixed methods) [59], CA	complex patients	NPs improve access for complex patients in an area short of primary care providers.
Collins (mixed methods) [60], UK	complex conditions	NPs treat a range from minor illness to complex conditions such as cancer.

Sources: *US* United States, *NL* Netherlands, *CA* Canada

Professionals: *NP* Nurse practitioner, *GP* General practitioner, *FP* Family physician, *NCM* Nurse care manager, *PCP* Primary care physician, *MD* Medical doctor

### Results of the narrative analysis

The understanding of the use of complexity was based on the broad context consisting of four aspects: patient population, setting, professionals and NP role taking information from the entire study into account as seen in Table 4. Excerpts of the respective text passages can be found in Appendix Table 5.

**Table 4:** Analysis of included studies with regard to complexity

Source	Patient Population	Setting	Professionals	NP role
Qualitative study design				
O’Brien et al. 2008, US	unspecific	unspecific	medical doctors & advanced practice nurses	NP is supervised and mentored by doctor
Parker et al. [24], AU	unspecific	unspecific: primary health care	GP & NP	Collaborator with GP in primary health care
O’Malley et al. [25], US	unspecific	PCMH	administrative staff, LPN, MA, NCM, NP, PA, physician, practice manager primary care experts, RN	Varies depending on practice, some are lead clinicians while others share care with physician
Van der Biezen et al. [26], NL	unspecific	unspecific: general practices, GPC	GP, managers, NP/PA	Substitute and supplement in general practice and substitute in out-of-hours care in GPC (Substitute and Supplement)
Lovink et al. [27], NL	older adults	unspecific: general practice & community	RN, NP & GP	Independent care providers, shared responsibility with GP, part of multidisciplinary team
Boman et al. [28], NO/FI	older adults	unspecific: primary health care	GP, NP & nurse	Autonomous role within scope of practice and linking role between nurses and physicians; patient and health services; and to evidence-based practice.
Côté et al. [29], CA	unspecific	unspecific: primary health care	GP & NP	Part of multidisciplinary primary care teams
Pelletier et al. [30], CA	unspecific	unspecific: primary health care	GP, RN & NP	Part of primary health care team
Quantitative study designs				
Ohman-Strickland et al. [31], US	diabetics	unspecific: family medicine practices	NP/PA & physician	Staff member
Everett et al. [32], US	unspecific	unspecific: outpatient practices	doctor & NP/PA	Primary care provider substitute to underserved patients with a range of disease severity
Subramanian et al. [33], US	diabetics	VHA	NP/PA & physician	Independent primary care provider
Yarnall et al. [34], US	unspecific theoretical panel	unspecific: ambulatory medical practices	NP/PA & physician	Member of primary care team
Chung et al. [35], US	unspecific	unspecific: GP practice	NP/PA & PCP	Part of medical practice
Mian et al. [36], CA	unspecific	CHC, family health teams, family practice units	FP, mental health worker, NP/PHCNP, social worker	FP and NP have interdependent roles in which NP is responsible for less complex care
Morgan et al. [37], US	unspecific	VHA	NP/PA & physician	Primary care provider
Donelan et al. [38], US	unspecific	unspecific: primary care practices	NP & physician	Practices with NPs describe them as a member of a collaborative pracitce.
Everett et al. [39], US	medicare diabetics	unspecific: various practices	NP/PA & physician	Supplement provider and usual provider (Substitute)
Everett et al. [40], US	medicare diabetics	unspecific: various practices	NP/PA & physician	Supplement provider and usual provider (Substitute)
Dahrouge et al. [41], CA	unspecific	CHC	FP & NP	Consultative care, in which they NPs are substitutes; or shared care, in which NPs are supplements(Substitute and Supplement)
Ku et al. [42], US	unspecific	CHC	lab staff, MA, NP/PA, physician, radiology staff	Member of staff with full, partial or restricted legal scope of practice
Kuo et al. [43], US	diabetics	unspecific: primary care in communities	NP & PCP	Care provider
Park [44], US	unspecific	PCMH and non-PCMH	NP/PA & physician	Complemental care provider
Reckrey et al. [45], USA	homebound	home-based primary care	administrative assistant, NP, physician, RN, social worker	Team member with flexible role
Marcum et al. [46, 47], US	chronically ill	unspecific: primary care group practices	NP/PA & PCP	Primary care provider
Raji et al. [48], US	medicare, chronically ill	unspecific: various practices	MD & NP	Independent primary care provider
Van der Biezen et al. [49], NL	unspecific	unspecific: GPC	GP & NP	Substitute in out-of-hours care
D’Afflitti et al. [50], US	unspecific	unspecific: general internal medicine practice	GP & NP	GP and NP teams co-managing medically complex patients
Yang et al. [51], US	diabetics	VHA	GP, PA & NP	Primary care provider
Morgan et al. [52], US	diabetics	VHA	NP/PA & physician	Primary care provider
Other study designs				
Fletcher et al. (mixed methods) [53], US	unspecific	VHA	NP & physician/MD/ doctor	According to NPs they practice autonomously with physician back up. According to physicians NPs are physician extenders. (Supplement)
Dierick-van Daele et al. (mixed methods) [54], NL	unspecific	unspecific: single practice, group practice, health centre	GP & NP	Collaborator in a team, role dependent on practice needs and incentives (Supplement)
Sustaita et al. (review) [55], US	unspecific	unspecific: various practices	NP & physician	Independent providers with a unique approach to health care who do not substitute physicians (Supplement)
Freund et al. (comparison) [56], NL	unspecific	unspecific: primary practices	GP, NP, extended role practice nurse, practice nurse/auxiliary	Part of primary care team focusing on minor illnesses.
Helms et al. (case study) [57], AU	unspecific	unspecific: bulk-billing health care cooperative	NP & GP	Collaborator, providing complimentary care
Bodenheimer & Bauer (perspective) [2, 58], US	unspecific	unspecific: primary care practice in the US	physician, NP, RN, PA	Approximation of that of a physician’s, primary care practitioner
Hunter et al. (mixed methods) [59], CA	unspecific	unspecific: rural community practice	HCP, healthcare leaders, NP, PCP	Collaborator with PCP and HCP (Supplement and Substitute)
Collins (mixed methods) [60], UK	unspecific	unspecific: out-of-hours care	GP & NP	Primary care provider

Sources: *US* United States, *NL* Netherlands, *CA* Canada

Settings: *VHA* Veteran’s Health Association, *PCMH* Patient-centred medical home, *GPC* General practitioner cooperative, *CHC* Community health clinicProfessionals: *NP* Nurse practitioner, *MD* Medical doctor, *GP* General practitioners, *FP* Family physician, *PCP* Primary care physician, *PA* Physician assistant, *HCP* Health care professionals, *LPN* Licensed practice nurse, *MA* Medical assistant, *NCM* Nurse care manager, *RN* Registered nurse, *PHCNP* Primary health care nurse practitioner

### Patient population

Patient populations are either specific or unspecific. Twelve studies included specific patient populations, two of which were older adults [27, 28], seven diabetics [31, 33, 39, 40, 43, 51, 52], two chronically ill [47, 48], and one home-bound [45], whereas twenty-five studies included unspecific patient populations consisting of general primary care patients.

In geriatric care NPs have been reported to be competent in performing assessments in adults requiring complex care, despite this however, the reality of introducing NPs into general practice may be that GPs focus on more complex geriatric care [27]. Alternatively, they may take on autonomous roles within their scope of practice managing complex geriatric care cases [28]. In diabetes care, complexity can be used to distinguish between medically complex patients, those with comorbidities, receiving GP care and socially complex patients, those effected by poverty, and consequences of dementia and depression, receiving NP care [40, 43]. Alternatively GPs may treat all complex cases while NPs provide supplemental care [39] or disease prevention measures resulting in improved care, for example in terms of adherence to diabetes care guidelines [31]. In certain Veteran Health Associations (VHA) NPs provided entire diabetes care independently with or without delayed physician supervision, which can be considered as the treatment of patients with high decision-making complexity [33, 51, 52].

When observing prescription patterns of GPs caring for chronically ill patients, it seems they care for more complex cases, because they prescribe more and newer medications compared to NPs. This assumption is derived from the fact that co-morbid patients require more medication [47]. This is in keeping with the concept of previous surrogate terms for complexity being comorbidity or multimorbidity [15]. On the other hand, the complexity of chronically ill patients with multiple chronic diseases may be a possible indicator for shared care involving both GPs and NPs equally, especially after recent hospitalization or new diagnosis [48]. In home-based care in which the patients’ medical as well as psychosocial needs must be met, the need for team-based models of care, in which NPs may care for the most complex patients, are promoted [45]. The use of complexity within unspecific patient populations was also broad, however, no clear trend was discernible.

### Setting

The settings are either specific or unspecific. Eleven studies include specific settings, two of which are Patient Centred Medial Homes (PCMH) [25, 44], three Community Health Centers (CHC) [36, 41, 42], five Veteran Health Associations (VHA) [33, 37, 51–53], and one home-based care setting [45], whereas twenty-six studies describe unspecific settings such as general primary care practices.

Both PCMHs, which are considered enhanced models of primary care aiming to improve quality, invoke better experiences and reduce costs [44], and CHCs, which are community-led, non-profit organizations delivering health as well as social and community services [41], are conceptualized with interprofessional teamwork in mind. In these settings, complexity may be used to allocate medically complex patients to GP care [42, 44] while NPs refer patients to GPs when conditions exceed their scope of practice or range of competence [36] and care for more socially complex patients to minimise consultations with GPs [41]. If however, NPs take on a lead clinician role they may care for all types of complex patients [25].

In the VHA, the largest integrated healthcare system in the US [37, 53], GPs initially casted doubt on the appropriateness of NPs substituting GPs and expressed the need for GP supervision, especially in complex cases [53]. And according to patient encounters, GPs did treat slightly more complex cases [37]. However, NPs increasingly fill similar roles as GPs, working independently and treating similarly complex patients [51, 52], albeit with some delayed GP supervision [33]. As mentioned above in a team-based model of care in a homebound setting, which predicates complexity based on medical and psychosocial needs, NPs may care for patients independently [45].

Similar to unspecific patient populations, the use of complexity is broad within unspecific settings and no clear trend is discernible.

### Professionals

Studies with more than two types of health professionals and only two types in collaboration are distinguished. Twelve studies include more than two health professionals [25–28, 30, 36, 42, 45, 51, 56, 58, 59], while only two health professionals were described in twenty-five studies [23, 24, 29, 31–35, 37–41, 43, 44, 47–50, 52–55, 57, 60].

In teams consisting of more than two types of health professionals, including other nurses, NPs are among the highest qualified, thus substituting GPs as lead clinicians providing complex care [25, 27, 28, 51] or managing complex patients within a shared care model [45]. In teams consisting of more than two health professionals and NPs are the only type of nurse or perform similar tasks to a nurse, they may treat less complex patients [26, 30, 56, 58] and improve overall access for them [59]. This depends on their legal scope of practice [42] and practice demands [36].

In teams consisting of only two health professionals NPs may treat less complex patients [23, 24, 29, 39, 43, 47, 49, 53–55] or more socially complex patients [40, 41]. This may lead to increased patient access to primary care [54], increased time for GPs to treat (medically) complex cases [34, 35, 44, 53–55], reduced referrals to specialists [35], and increased patient outcomes [31]. On the other hand, NPs may also treat complex patients themselves [32, 33, 37, 48, 50, 52, 57, 60].

The question of which professional treats complex patients may not be answered the same way among professionals themselves. Both professionals self-reportedly treat complex patients. However, not many GPs report that NPs treat complex cases [38].

### NP roles

NP roles are either described within a distinct model of care or they are unspecified. In seven studies a distinct model of care [26, 32, 39–42, 49] is illustrated, whereas in thirty it is not.

Models of care involve NPs in the role of either a usual provider, a substitute or a supplement. NPs working as usual providers manage their own patient panels independently. Similarly, NPs functioning as substitutes also manage their own patient panels, however have the possibility to access consultations with a GP similar to any other GP working in a group setting. NPs working as supplements have almost no overlapping tasks with GPs, and thus provide supplemental care. An influential factor on the role of a NP is legislative scope of practice, which may range between full, partial, or restricted scope and defines the range of services provided by a NP.

The evidence shows that NPs as usual providers may treat socially complex patients [40]. Similarly as substitutes they may care for more socially complex patients to minimise consultations with GPs on medical complexity [41] or they may be substitutes only for minor ailments [49]. Alternatively, they may treat under-served patients, who do not differ in medical complexity compared to GPs’ patient panels [32]. Despite potentially full scope of practice, NPs may function as supplements and are less involved in complex care [42]. This scenario may result in as good as or better results in patient care than exclusive GP care [39].

The use of complexity is broad where there is a distinct model of care and, where there is a lack thereof. Furthermore, it is noteworthy that NPs substituting GP tasks is not synonymous with them treating an equivalent patient panel.

## Discussion

### Main findings

The use of complexity as a factor for task allocation is generally inconsistent. However, trends were recognized: Complexity is used to describe patients, their needs, and health professionals’ tasks. The understanding of the use of complexity as a factor for task allocation between NPs and GPs is based on the patient population (specific vs. unspecific), the setting (specific vs. unspecific), the numbers of health professionals involved (two vs. more than two), and the NP role (distinct model of care vs. no model). Despite similarities in these areas, the tasks which NPs take on range from minor to complex. So for example, a NP’s role may be described as that of a GP substitute, yet only substitute non-complex care, or alternatively take on an entire patient panel with the same complexity as a GP. However, a distinction between medical and social complexity is noticeable throughout all included literature, with a tendency towards GPs treating more medical complexity, while NPs treat more social complexity.

### Interpretation & comparison with existing literature

Allocating tasks according to complexity and the professionals’ ability to deal with said complexity is reflected in Kernick’s continuum: This is in keeping with some results of the included studies: complexity is used to allocate patients to health professionals according to their educational ability to treat complex cases [25, 56]. However, given that the included studies originate from countries in which, the NP profession is being developed or is well established and mostly includes an education on Master’s level, Kernick’s continuum is not evident in all practice settings. Hence, when practitioners are considering which tasks to allocate to NPs, it may be indicated to have thorough knowledge of their educational ability, which may define to what extent or in which context complex tasks can be performed.

Similarly the way in which complex systems are displayed in the Cynefin Framework is highly relevant for many of the included studies in primary care. An adaptation of the framework states that all four systems are represented in primary care. GPs are said to manage the simple and complicated systems, including performing therapeutic procedures and prescribing medication. NPs are said to manage complex systems, including the management of chronic illness by supporting and empowering patients to change attitudes, beliefs, and behaviors [61]. This is in keeping with findings in this systematic review: assuming GPs manage medical complexity in simple and complicated systems and NPs manage social complexity in the complex system, i.e. the distinction between medical and social complexity is recognisable. Here it may be of value for practitioners to consider in which system they consider themselves to be active and how tasks can be divided accordingly.

An influential factor on the NP role is legislative scope of practice, which varies largely among countries and regions and informs NP training as well as competencies. However, broadening the legal scope of practice and hence the educational curriculum are not the only steps needed for NPs to care for complex patients. As seen in an example from the Netherlands, where NPs are allowed and able to care for patients with complex conditions, might not do so based on the conceptualisation and traditions of the practice setting [26]. In Indicating that changes in extended areas are needed for NPs to fulfil their potential in practical settings. This observation is supported by Weiland who reports that political, social and professional changes need to take place for NPs to meet society’s health care requirements [62]. Therefore, it is not merely an issue of legislative adjustment, but a matter of developing practice dynamics in which practitioners play a vital role. A further concern is the reimbursement system in place, which may encourage or discourage the employment of NPs in primary care [63]. Tasks may be assigned to a professional based on remuneration to the practice rather than according to actual skills according to a review on facilitators and barriers influencing GP and NP teamwork [64].

### Implications

To further clarify the allocation of complex care within interprofessional teams, role descriptions for all health professionals in primary care need to be developed given the country’s health care system and legislative framework. Factors which could be integrated and clarified in a role description are job titles, training, and tasks which could be determined with a functional job analysis [65]. This may be a vital step towards redesigning the system and changing the culture of team work which is evidently needed given the introduction of NPs in primary healthcare [66]. Whether a common yet individually adaptable model for multiple countries would be a viable option is unclear from these results.

Further, role understanding can be encouraged to allocate complex tasks appropriately. This, along with a collaborative work environment, can be facilitated through interprofessional education [67] which “occurs when two or more professions learn about, from and with each other to enable effective collaboration and improve health outcomes” [68]. Additionally, clearer regulations with regard to scope of practice, reimbursement and accountability could enhance skill-mix by increasing NP participation in primary care [63]. As shown in the included literature, various mixed models of care in which roles are mutually understood and skills are appropriately distributed according to regulations can lead to increased efficiency of patient care [54, 59].

Future research could include an overview of university curricula and role descriptions in practice in various countries. This may lead to more knowledge regarding the possibility of creating an over-reaching NP role description including concrete references to the allocation of complex medical and social care, which could be applicable across countries. Furthermore, researchers should determine if a clear allocation of complex care is associated with higher job satisfaction. We also anticipate that clarity could improve role identity and self-confidence among NPs, especially, if inexperienced GPs see the potential value of NPs to the team and support their development [69]. Lastly, improving knowledge about complexity might allow policymakers to develop more transparent and fairer remuneration systems for NPs and GPs.

### Limitations

First, the data-derived extraction sheet might have introduced a risk of extraction bias. For example, education was not explicitly represented as a criterion upon which the complexity of tasks could be allocated. However, using this method allowed an extraction process, which remained true to the data at hand, hence, it is to be understood that insufficient information regarding educational level was given in the included literature.

Second, heterogeneity in terminology led to included studies predominantly originating from the US. The term, as well as the profession of NPs, were born in the US in the 1960s, thus an abundance of literature and experience are available. Even though the term NP is well known, other countries have used different terminology. Heterogeneity in terminology may have also led to the exclusion of forward cited literature. In some original sources, the concept of complexity was not explicitly featured, and the citing authors interpreted a described situation as being “complex”. Additionally, restricting the literature search to English and German articles may have resulted in missing publications. Furthermore, we may have missed historical concepts of complexity that were not mentioned in any article that was published within our limited time frame of 13 years.

Third, it can be assumed that tasks are shared among GPs and NPs in primary care settings for which the methods are not published in scientific journals but in policy documents, particularly in less developed countries. Hence, these methods are not visible in the presented results.

Lastly, the lack of restriction on study design meant that heterogeneity in the types of included studies occurred. Consequentially the results are not directly comparable. However, this was not considered a major issue given that the way the search was structured, the aim was to find in which context the word “complex” was used. Hence, the methodological soundness of the individual studies has limited bearing on the statement referring to complexity.

## Conclusion

This narrative review delivers an overview of the varied use of complexity and can be used as a point of reference when practitioners are seeking methods for task allocation in a collaborative primary care setting. Complexity has a broad and inconsistent use as a factor for task allocation. However, the findings show, that complexity as a concept is prominent in primary care not only because of increasing rates of chronic illness in an ageing population but also because collaborative practice is on the rise. There is a slight trend towards NPs treating socially complex patients and GPs focusing on medically complex cases. Furthermore, complexity is used to describe patients, their conditions and professional’s tasks. Hence, it may make sense to distinguish a “complex patient” or “complex condition” in terms of medical or social complexity to allocate tasks between GPs and NPs. Task allocation based on complexity can be observed based on patient populations, the setting, the involved health professionals and the roles they take. So, not only can the complexity of the patient and their condition be assessed when allocating tasks but also how many and what types of health professionals are available to provide care. This means that a NP may be one of the highest qualified and therefor may take on a complex caseload, similar to that of a GP or may share complex care according to the given professional abilities. To a large extent however, task sharing according to complexity is also influenced by overreaching legal frameworks which in turn influence education, competencies and team-work culture within practices.

## Data Availability

Not applicable

## Appendix

**Table 5:** Complexity Quotes

Source	Quotes
Qualitative study design	
O’Brien et al. 2008, US	“The physician often has a more in-depth background in some of the **complex** medical issues and takes the leadership in that.”
Parker et al. [24], AU	“Some consumers saw that seeing a nurse practitioner for minor ailments would ‘free up the GP’ to deal with more **complex** medical concerns, but was also connected to not wasting the GP’s time.” “These values and skills provided the basis for the focus group respondents’ approval towards appropriately trained nurse practitioners being acceptable primary health care providers, in particular for minor ailments and less **complex** conditions.”
O’Malley et al. [25], US	“Primary care physicians provide comprehensive whole-person care, including preventive, acure and **complex** care needs.” “Nurse care manager (relatively new role, most often filled by RN): works with **complex** patients on care plan, goals, education; monitor periodic labs and results.” “Offloading of routine tasks to MAs and LPNs resulted in increased job satisfaction for physicians in several practices, who could instead focus on patients’ more **complex** and personal needs.”
Van der Biezen et al. [26], NL	“Some GPs wanted to employ the PA/NP in order to replace a GP, to expand the number of patients in their practice or to create job opportunities for their own professional development (e.g. focussing on more **complex** patients, more time for study or ancillary activities).” “[...] we initiated to work with NPs to meet the increase in patients so that GPs can focus on the **complex** patients.” “As a consequence of the PA/NP treating the less **complex** patients, all GPs expected a difference in their own caseload. While some GPs considered this an opportunity for their own professional growth and enhancing job satisfaction, others feared a more **complex** caseload. This included a fear of losing routine in treating minor ailments or an increased work pressure due to more **complex** complaints during surgery hours.”
Lovink et al. [27], NL	“NPs were reported to be competent to screen older adults with **complex** care needs.” “The introduction of NPs, PAs and RNs changed the role of GPs from a more clinical expert role for all patients to a more coordinating role with focus as clinical expert on the more **complex** patients. Positive perceived effects were that the workload for the GPs became lower, that their practices could be larger and that they had more time to focus on the more **complex** patients. Negative perceived effects were that the GPs had less patient contact and less freedom because they should be available for the NP, PA or RN and that the GPs only had consultations for **complex** patients increasing the caseload as NPs, PAs and RNs only had consultations for less **complex** patients.”
Boman et al. [28], NO/FI	“The GNPs were also envisioned to take on a linking role between patient and health services, taking on a case management role especially for patients with comorbidity and **complex** care needs.”
Côté et al. [29], CA	“For many physicians, collaboration with PHCNPs has meant that their own case loads have included more medically **complex** patients, while less medically vulnerable patients are being directed toward the PHCNPs.”
Pelletier et al. [30], CA	“Respondents felt that PHCNPs enable family physicians to manage more chronically ill patients with more **complex** health situations. However, this redistribution was perceived by some to increase physicians’ workload because physicians would then be dedicating more of their time to **complex** cases.” “Most respondents observed that PHCNPs devote most of their time to a highly diversified clientele and to acute care for a great variety of **minor** medical problems.”
Quantitative study designs	
Ohman-Strickland et al. [31], US	“For instance, when a practice uses either PAs or NPs, the practice’s overall performance may reflect the distribution of patients to clinicians. **Complex** patient cases may be assigned to physicians, more routine or acute cases may be assigned to PAs, and cases requiring a more preventive approach may be assigned to NPs. Theoretically, this could lead to more efficient and individualized patient care.”
Everett et al. [32], US	“Populations served by PA/NPs and doctors differ demographically but not in **complexity**.”
Subramanian et al. [33], US	“Most NPs and some PAs in the VHA system practice more or less independently, or under delayed physician supervision, and manage patients requiring high levels of decision-making **complexity**.”“We can say that given the expanding role of midlevel providers in delivering primary care to **complex** patients, we need to better understand whether differences in BP treatment change by provider type at a single visit lead to long-term differences in BP management and control.”
Yarnall et al. [34], US	“Additional nonphysician clinicians - including physician assistants (PAs) and nurse practitioners (NPs) - can expand the amount of time available for patient care and allow physicians to focus on the most **complex** medical care issues.”
Chung et al. [35], US	“PCP’s with NP-PA were found to have a greater likelihood of treating patients with **complex** conditions instead of referring them to specialists.” “PCPs with NP-PA were also found to provide appropriate care to the **complex** patients. These findings indicate that NP-PA enable PCP to concentrate on patients with more **complex** conditions thus reducing the number of referrals.” “The use of NP-PA is viewed as being causally linked to increases in treating patients with **complex** conditions.”
Mian et al. [36], CA	“Referrals of clients from FPs to PHCNPs may reflect both FPs’ reliance on NPs’ unique competencies and “value-added” skills in communication and employment of NPs as substitution for less **complex** care [as supported by previous evidence].”
Morgan et al. [37], US	“Nurse practitioner and PA patients had slightly lower DCG **complexity** scores than physician patients (physicians, 0.89; NPs, 0.84; PAs, 0.82) [but] can be considered similar across the three provider groups.” “The finding of only small differences in this measure of patient **complexity** challenges the prevailing notion that NPs and PAs see patients who are less medically **complex** than those cared for by physicians.” “Within encounters for established patients, physicians staffed slightly more visits towards the more **complex** end of the spectrum than did NPs or PAs.” “Overall, NPs, PAs, and physicians filled similar roles in VHA primary care clinics, although there were some differences in patient **complexity** and purpose of visits.”
Donelan et al. [38], US	“Clinicians who agreed with this statement were asked to identify the types of services that were primarily handled by physicians: 43.8% of physicians and 21.1% of nurse practitioners cited care for more **complex** cases [...].”“Although physicians and nurse practitioners differed significantly on most items, the majority of the two groups reported that most services were performed by both providers, with the exception that only 28.3% of physicians agreed that nurse practitioners provided services for **complex** chronic conditions that were complicated by coexisting conditions or were not yet well controlled.”
Everett et al. [39], US	“Panels with PAs or NPs as supplemental providers that provided care to at least one patient with a risk score of 2.0 or greater (that is, twice the average predicted use of services for older patients) were categorized as providing care to highly **complex** patients.” “Patients with supplemental PAs or NPs who did not treat highly **complex** patients consistently experienced similar or better outcomes, compared to patients receiving physician-only care. In contrast, patients with supplemental PAs or NPs who did treat highly **complex** patients experienced several worse outcomes, again compared to patients receiving physician-only care.” “For example, if the primary goal is more frequent testing of glycemic control, then the addition of supplemental PAs or NPs who do not treat highly **complex** patients but who do deliver care for chronic conditions might be appropriate.”
Everett et al. [40], US	“Panels with PA/NPs as usual providers appear to have a higher proportion of socially **complex** patients, when defined according to poverty (Medicaid), disability, and co-morbid dementia and depression. [...]. In contrast, the clinical **complexity** of patient panels appears similar regardless of usual provider type [...].” “The probability of patients having a visit with a supplemental physician (5–48%) is significantly higher on panels with PA/NPs as usual providers [...]. [...]. PA/NPs may not have the clinical expertise to meet all the medical needs of older, **complex** patients with diabetes and refer the patient to physicians more frequently. Alternatively, it could be a deliberate approach to ensure participation of both providers in the PA/NP-physician dyad and adequate access to care for socially **complex** patients.”
Dahrouge et al. [41], CA	“Patients who received care in the FP model of practice had more **complex** medical conditions (cardiovascular disease, mental illness, lung disease, and diabetes) and more annual visits.” “To maximize NPs’ ability to care for their own patients with minimal consultation with FPs, CHCs might have used intake questionnaires to determine whether an incoming patient would be assigned to an NP (less medical **complexity)** or FP (greater medical **complexity).** Nurse practitioners who found themselves caring for more medically **complex** patients were probably obliged to have FPs provide care that they were unable to provide themselves, potentially explaining the finding that shared care patients had characteristics intermediate to the FP care and NP care patients.” “Compared with FPs, NPs saw patient panels that were less medically **complex** but more socially **complex.”**
Ku et al. [42], US	“We measured productivity as the number of weighted medical visits per center in 2012. Weighting is important because medical visits vary in the **complexity** of care required, which may also influence the type of staff involved.” “This signals that weighting increases the apparent contribution of physicians and decreases the apparent contribution of advanced-practice staff [including NPs], which suggests that physicians are more involved in **complex** visits, compared to the advanced- practice staff.” “Our analyses support evidence that physicians tend to be more involved in care for patients with **complex** conditions, compared to nonphysician medical staff [including NPs].”
Kuo et al. [43], US	“The frequent specialist consultations suggest that NPs recognize limitations in their training when caring for medially **complex** individuals with multiple comorbidities.” “[...] NPs may deliver care to healthier, less medically **complex** individuals than PCPs, but comparing results from unmatched analyses shows that nonpooling propensity score matching reduced the differences between the two groups on several measurements for disease and medication management.”
Park [44], US	“The use of NPs and PAs may continue to accelerate with the growth of PCMHs because it allows them to accommodate patients and enable care coordination, thereby ensuring physicians more time to devote to **complex** cases.”
Reckrey et al. [45], US	“Team-based models of care are an important way to meet the **complex** medical and psychosocial needs of the homebound.” “It was expected that the nurse practitioner would help most with straightforward cases, freeing physicians to address **complex** care situations when they returned to the office, but the nurse practitioner instead took an enhanced role in the management of the most-**complex** patients on the Team Approach physician panels.”
Marcum et al. [46, 47], US	“Our findings are consistent in that PCPs were more likely than NPs and PAs to prescribe to older patients, who often take multiple medications due to chronic co-morbidity. Given an older, more **complex** patient panel, PCPs may be more likely to prescribe from a broader prescription armamentarium, including newly approved drugs.”
Raji et al. [48], US	“The NP-MD team model may best serve the needs of the switch group patients whose health conditions have become more **complex** following their recent hospitalizations or new diagnoses.”
Van der Biezen et al. [49], NL	“Moreover, in the current study the NPs were primarily responsible for treating **minor** ailments. The **complexity** of tasks can differ between regions and countries.”
D’Afflitti et al. [50], US	“[...]A full-time NP saw patients for 6 half-day clinic sessions. [...]. Such care included phone calls to patients for chronic disease management, test result follow-up, care coordination with specialists, and outrreach to medically and socially **complex** patients in an effort to keep them engaged in care.”
Yang et al. [51], US	“Primary care nurse practitioners, physician assistants, and physicians at the Veterans Health Administration care for diabetic patients with similar medical **complexity**.”
Morgan et al. [52], US	“These results combine with our previous findings to provide additional support for the use of PAs and NPs in the primary care of **complex** patients.” “This study, combined with previous findings that diabetes care quality in the VA did not differ by primary care provider type, suggests that NPs and PAs can effectively manage primary care for medically **complex** patients with diabetes without increasing total care costs.”
Other study designs	
Fletcher et al. (mixed methods) [53], US	“Potential benefits to VHA from the use of NPs include being able to provide care to more patients at the primary care level, and providing additional time for physicians to spend with more **complex** patients.” “One rationale for using NPs in primary care is that physicians are more readily available to handle **complex** cases.” “In contrast, most of the physicians who commented on the NPs’ competence tended to think that NPs were not qualified to manage a panel of **complex** patients without constant, direct MD supervision.” “Most physicians emphasized the importance of NPs working within a limited scope of practice or caring for simple cases. “NPs are always the best working under direct supervision with doctors. They can be utilized best as case managers or seeing low **complexity** patients.” “Fifty-eight (78%) of MD respondents agreed that NPs are well integrated into “our” practice setting, but 42 (58%) agreed that NPs care for patients who are too **complex** and 38 (52%) affirmed that NPs are often assigned patients too complicated for the NPs’ abilities.” “There appears to be a fine line between NPs’ desires for autonomy and being pushed beyond their scope of practice in a large system with many **complex** patients.”
Dierick-van Daele et al. (mixed methods) [54], NL	“The NPs should assess, diagnose and treat a specified set of common complaints. Therefore, they needed to possess medical knowledge and use practice guidelines on **minor** health problems derived from the Dutch College of General Practitioners.” Quote from GP: “We share home visits of patients with **complex** care (palliative care). It is comfortable and professional, when I do not have to do this all by myself. I feel that I have a new partner in these situations.” “As NPs mainly treat patients with common complaints this might also lead to GPs having more time for patients with chronic diseases or multi-morbidity.”
Sustaita et al. (review) [55], US	“Many physicians feel overwhelmed with routine responsibilities, such as completing forms, taking patient telephone calls, performing physical exams, and attending to urgent unscheduled appointments. An NP can take ownership of these responsibilites so that the physician may spend more time with higher-acuity or more **complex** patients.”
Freund et al. (comparison) [56], NL	“Area B (nurse practitioner): Clinical diagnosis and treatment of less **complex** presentations (minor illnesses) and also chronic care management”
Helms et al. (case study) [57], AU	“The PHC-NP has his own caseload but also receives referrals from GP, nursing and allied health team members within the co-op, for the opinion and management of **complex** cardiovascular conditions and lifestyle modification interventions, as this is his area of primary expertise and interest.” “It was also felt that GP productivity increased because the PHC-NP would manage clients with **complex** chronic diseases, who take a great deal of time and attention in their management.”
Bodenheimer & Bauer (perspective) [2, 58], US	“Physicians will probably focus on diagnostic conundrums and lead team caring for patients with **complex** health care needs.”
Hunter et al. (mixed methods) [59], CA	“Introduction of the NP role improved access to care in an area short of primary care providers, with 817 previously unattached patients added to the NP’s caseload. Access was also improved for some **complex** patients.”
Collins (mixed methods) [60], UK	“The variance and potential **complexity** ranges from **minor** illness to more **complex** conditions such as terminal care.”

Sources: *US* United States, *NL* Netherlands, *CA* Canada

Settings: *VHA* Veteran’s Health Association, *SDNPC* Sudbury district nurse practitioner clinics, *CHC* Community health centre, *PCMH* Patient-centred medical homeProfessionals: *NP* Nurse practitioner, *MD* Medical doctor, *GP* General practitioner, *RN* Registered nurse, *MA* Medical assistant, *LPN* Licensed practice nurse, *PA* Physician assistant, *PCP* Primary care physician, *FP* Family physician, *PHCNP* Primary health care nurse practitionerOthers: *BP* Blood pressure, *PHC* Primary health care, *DCG* Diagnostic cost groups.

## Abbreviations

CHC: Community Health Centre
GP: General practitioner
MeSH: Medical subject headings
NP: Nurse practitioner
PCMH: Patient centred medical home
VHA: Veterans Health Association
